# High genetic diversity of spider species in a mosaic montane grassland landscape

**DOI:** 10.1371/journal.pone.0234437

**Published:** 2020-06-08

**Authors:** Jason L. Botham, Charles R. Haddad, Marieka Gryzenhout, Vaughn R. Swart, Emile Bredenhand

**Affiliations:** 1 Department of Zoology and Entomology, University of the Free State, Bloemfontein, Free State, South Africa; 2 Department of Genetics, University of the Free State, Bloemfontein, Free State, South Africa; 3 Department of Zoology and Entomology, University of the Free State Qwaqwa Campus, Phuthaditjhaba, Free State, South Africa; National Cheng Kung University, TAIWAN

## Abstract

Gene flow and genetic variation were examined within and among populations of five of the most common spider species in shrublands of the mountainous Golden Gate Highlands National Park (GGHNP), South Africa. These species included three active hunters, *Dendryphantes purcelli* Peckham & Peckham, 1903 (Salticidae), *Pherecydes tuberculatus* O.P.-Cambridge, 1883 (Thomisidae) and *Philodromus browningi* Lawrence, 1952 (Philodromidae), and two web-builders, *Neoscona subfusca* (C.L. Koch, 1837) (Araneidae) and a *Theridion* Walckenaer, 1802 species (Theridiidae). A total of 249 spiders (57 *D*. *purcelli*, 69 *N*. *subfusca*, 34 *P*. *browningi*, 56 *P*. *tuberculatus* and 33 *Theridion* sp.) were collected and analysed from six shrubland localities in the park. Analyses of sequence variation of the mitochondrial cytochrome oxidase c subunit I (COI) gene for each species revealed relatively low nucleotide diversity (π < 0.0420) but high genetic diversity (Hd > 0.6500) within populations for all species, except *P*. *tuberculatus*. Genetic differentiation was also noted to differ between species, with only *P*. *tuberculatus* indicating very large divergence (Fst > 0.2500). These results were reflected by gene flow, with *D*. *purcelli*, *N*. *subfusca* and the *Theridion* sp. estimated as experiencing more than one disperser per generation. Overall, highest gene flow was found in the two web-building species, indicating possible high dispersal ability of these spiders in the GGHNP. Additionally, constructed phylogenies indicated possible cryptic speciation occurring in the majority of the investigated species. Our current results indicate that the five investigated spider species were able to maintain gene flow between shrubland populations within the GGHNP to some degree, despite the mountainous landscape. However, further analyses incorporating additional molecular markers are needed to properly determine the extent of genetic diversity and gene flow of these species within the GGHNP.

## Introduction

Genetic diversity and gene flow between fragmented faunal populations is warranting an ever-increasing focus from a conservation perspective [1–2]. Ensuring phenotypic and genotypic similarities between populations of the same species relies upon essential connectivity, particularly across wide distribution ranges [3]. Habitat fragmentation, giving rise to remnant woody vegetation patches in a grassland matrix, produces variable and unique challenges to faunal lifestyles [4–5]. Dispersal between such fragmented habitats is often dependent upon the distance of individual habitats in relation to native vegetation [6], with connectivity in and among populations being a direct consequence of taxon dispersal ability [7–10].

While mountainous regions provide a variety of habitats over a relatively small area, they often act as geographical barriers, limiting the extent of dispersal and gene flow [11–14]. The Drakensberg mountain range of South Africa is one such example, with responses of gene flow and genetic diversity differing among certain taxa [15–18]. However, corresponding data pertaining to terrestrial species across this mountain range remains limited. Mountains, rivers and oceans are regarded as the main topographical factors associated with gene flow and long-term barrier effects [19–20]. In spite of this, while elevation and mountain ridges show an effect on terrestrial population connectivity [12,21–23], mountains may not act as absolute barriers, instead forming more permeable filters [24]. This, in turn, solicits different responses and variations in genetic structures spatially for different taxa.

Limitations to population homogeneity, in the form of incomplete or fragmented phenotypic and genetic variability, can occur due to physical and/or behavioural barriers [25–26]. Those species with an aerial dispersal ability often possess a more homogenous genetic structure over a large geographical range compared to their substrate-bound counterparts, particularly spiders [10,27–29]. While large numbers of spider species possess conserved morphological characteristics, highly conserved morphology in cryptic species is often associated with strong genetic differentiation [30]. Additionally, phenotypic plasticity in the same spider species, due to the influence of local environmental factors as well as climate change, may be observed [31–32]. Investigating gene flow over a smaller geographical range, as to provide a basis for further genetic and taxonomic studies, becomes important in this regard. Concurrently, accurate delineation of species boundaries is fundamental in ecological and evolutionary studies, particularly in the assessment of biodiversity and the identification of areas of conservation priority [18]. The presence of national parks and reserves across the Drakensberg provides areas to investigate such factors before application on a larger geographical scale.

One such locality is the Golden Gate Highlands National Park (GGHNP), situated in the foothills of the Maluti Mountain Range (28°30’ S, 28°37’ E). Covering an area of approximately 340 km^2^, and possessing an elevational range of approximately 1600–2900 m a.s.l. [33], it is the only National Park in the Free State Province of South Africa. The park itself is classified as a montane grassland [34], with a variety of fragmented and insular shrubland patches occurring throughout the grassland matrix, most prominently in ravines and gorges [35].

Here we report on the first study to determine genetic diversity and gene flow of five of the most ubiquitous spider species in shrublands of the GGHNP. The selected species include *Dendryphantes purcelli* Peckham & Peckham, 1903 (Salticidae), *Neoscona subfusca* (C.L. Koch, 1837) (Araneidae), *Pherecydes tuberculatus* O.P.-Cambridge, 1883 (Thomisidae), *Philodromus browningi* Lawrence, 1952 (Philodromidae), and a *Theridion* Walckenaer, 1802 species (Theridiidae). Regarding the identified *Theridion* sp., it was not possible to accurately identify individuals to species level, however, all specimens were morphologically identical and considered to be part of the same species. The five investigated taxa encompass a range of different dispersal behaviours and foraging strategies (active hunting or web-building), with aerial dispersion, via wind-mediated dispersal (“ballooning”) of immatures, being a possible primary factor.

While ballooning allows for long-distance dispersal of spider species [36–37], it is often random and dependant on environmental conditions and surrounding topography [37–38], alongside an individual’s predisposition and motivation [39]. However, lifestyle can also modulate the propensity of ballooning in spider species, due to specialisation and feeding behaviour [40]. It is also considered that web-building spiders may have more opportunities to balloon, as they have well-developed silk production [41]. Additionally, while dispersal is predominantly restricted to juveniles in larger spider species [42], small-bodied species are able to initiate ballooning regardless of developmental stage [40,43]. Consequently, gene flow and dispersal capability may differ from species to species, affecting genetic diversity and population heterogeneity. Furthermore, while ballooning allows for quick dispersion and colonisation of new areas, its effectiveness in maintaining gene flow in highly fragmented landscapes may become limited due to its, largely, one-way movement [44–45].

In this study, we aimed to determine population structure to infer dispersal capability of the five selected spider species. To achieve this, molecular approaches involving *mt*DNA sequences of cytochrome c oxidase subunit I (COI), to determine phylogeographic diversification fragments, were applied on selected populations of these five spider species in the GGHNP. Due to their differing lifestyles, we hypothesised that the web-building spiders (*N*. *subfusca* and the *Theridion* sp.) would experience higher gene flow compared to the vegetation-dwelling active hunters (*D*. *purcelli*, *P*. *tuberculatus* and *P*. *browningi*) which possess more mobile lifestyles but tend to be more strongly substrate-dependent.

## Materials and methods

### Ethics statement

All animal ethics and collection permits were obtained from South African National Parks (SANParks) Scientific Services (Permit no. BOTJ1406) and the University of the Free State Ethics Board (Clearance no. UFS-HSD2017/0084).

### Study species

Due to the differing lifestyles, behaviour and morphology associated with the five investigated spider species, a short taxonomic description of the genera and species, along with their general distribution and vegetation commonly inhabited in South Africa, is given.

*Dendryphantes* C.L. Koch, 1837 is a widely distributed genus of jumping spiders (Salticidae) with a near global distribution, with the exception of Australia and Southern Asia [46]. More than 50 species have been described, nine of which are known from the Afrotropical region. The general appearance of *D*. *purcelli* in both sexes (S1A and S1B Fig) includes a flat, brown, oval carapace [47]. The abdomen is predominantly greyish-beige (slightly paler in females), with traces of a brown herringbone/chevron pattern and a pair of rounded brown spots at the midpoint. Individuals are relatively small, being on average 4.0–4.5 mm in length [47–48], and maintain a plant wandering, free-living, asocial lifestyle. This species has been collected from foliage of a variety of shrub and grass species across South Africa [47,49–50], and its distribution extends to the Western Cape Peninsula [51–52]. While lack of records exists for *D*. *purcelli*, large numbers of jumping spider species are known to balloon as juveniles [53–54]. Those species belonging to the genus *Dendryphantes* have also been postulated to make use of this dispersal method [55–56].

The 123 species of *Neoscona* Simon, 1864 (Araneidae) occupy a mostly pantropical distribution [46,57], with the investigated orb-weaver *N*. *subfusca* being widely distributed throughout the Afrotropical region, particularly in the Savanna and Grassland biomes of South Africa [49–50,52]. Individuals have been collected from a variety of grass, herb and foliage types across a number of habitats [58–60], where they maintain a preferential solitary [61], plant-dwelling lifestyle due to their construction of symmetrical orb-webs. *Neoscona subfusca* is often yellow to light brown in colouration, with a covering of white hairs and darker abdominal markings (S1C and S1D Fig) [62]. The abdominal dorsum is light, with a folium-like pattern, which is variable between individuals. Individuals range from 5 to 8 mm in length. Due to the web-building lifestyle of orb-weavers, ballooning is a common occurrence in *Neoscona* species [63–64]. Two infraspecific taxa of *N*. *subfusca* have been recorded, consisting of the subspecies *Neoscona s*. *alboplagiata* Caporiacco, 1947 from Tanzania and *Neoscona s*. *pallidior* (Thorell, 1899) from Bioko [46]. However, literature is unclear why Grasshoff [62] never synonymized them with *N*. *subfusca* in his revision of the Afrotropical fauna, but possibly he never examined their types and refrained from synonymizing them.

*Pherecydes* O.P.-Cambridge, 1883 is a genus of crab spiders (Thomisidae), comprised of seven species largely restricted to the Afrotropical region [46], and a very wide distribution in South Africa [52]. Both sexes of *P*. *tuberculatus* are structurally similar, with differences only occurring in colouration (S1E and S1F Fig) [65–66]. Individuals of this species are dark grey to brown, with males possessing an often mottled, darker carapace suffused with yellowish-white. Abdominal colouration is often a dull greyish-white with yellow tinges, and is marked with yellow-brown and dark brown (very dark ventrally for males). Body length of individuals range between 3–5 mm. While possessing a free-living, wandering lifestyle, and occurring on a variety of vegetation [49–50], individuals of *P*. *tuberculatus* were found to prefer “Wild Olive” (*Olea europaea* L. ssp. *africana* (Mill.) P.S. Green) above three other tree species studied [60]. Additionally, there is no record of sociality occurring in the genus *Pherecydes*. As many foliage-dwelling crab spider species are known to balloon during infancy [67–68], it can largely be assumed that *P*. *tuberculatus* employs the same method.

While superficially similar to the “true” crab spiders (Thomisidae), individuals of Philodromidae often have reduced true setae on their bodies and lack congruent eye tubercles [69]. The genus *Philodromus* Walckenaer, 1826 is the most common of the philodromid crab spiders (over 250 species), and is cosmopolitan with a largely Holarctic distribution, with a few species occurring in Africa and Australia [46]. Individuals of *P*. *browningi* are distinctly flattened and clothed in soft recumbent setae, and are generally large in size (carapace length average 8.5 mm) (S1G and S1H Fig) [70]. Body colouration is often reddish-brown with cream markings, and legs are generally pale with dark spots and bands. This species is also widely distributed in South Africa, being commonly found wandering on bushes, tall grass, herbage and the trunks of trees [49–50,52]. In *P*. *vulgaris* (Hentz, 1847), large numbers of individuals occurring in close proximity to one another have been reported, typically forming “pseudo-flocks” during periods of winter quiescence [71]. However, it is unknown whether individuals of *P*. *browningi* share this behaviour. With regards to dispersal ability, it can largely be assumed that *P*. *browningi* juveniles balloon, based on reports of its occurrence in other *Philodromus* species [72–73].

Species belonging to the tangle-web spider genus *Theridion* (Theridiidae) are often classified as gumfoot-web builders, whereby a three-dimensional web, consisting of a central area with or without a retreat, is constructed [50]. This genus contains nearly 600 described species around the world, with approximately 14 species recorded in South Africa [46,52]. Unfortunately, most of the South African species have never been illustrated or redescribed [46], making the identification of *Theridion* to species level extremely challenging. Further, the genus is a classical “dumping-ground” in spider taxonomy, with several generic synonyms, many species transferred to other genera, and no attempt to resolve its composition on a global scale yet [46]. As our investigated species clearly does not belong to any of the other theridiid genera recorded from South Africa to date [52], most of which are clearly distinguishable, we placed this species in *Theridion* based on the generic definition of Levi & Levi [74]. As it was not identified to species level, a general overview of the genus’ morphology and its distribution is given. *Theridion* individuals often possess a globular abdomen with cream or greyish-brown colouration (S1I and S1J Fig) [74]. Additionally, a distinct, notched median pattern extends along the abdominal dorsum, ranging from grey-white to brownish-red. Individuals are small, ranging in size from 3 to 5 mm. Although the genus is widely distributed throughout South Africa, confirmed species records remain incomplete for most taxa [49–50,52], due to the poor state of taxonomic knowledge. Subsocial behaviour in a related species, *T*. *pictum* (Walckenaer, 1802), has been well documented [75], alongside several other cooperatively social species in the same family, including a few species of *Theridion* (e.g. [76–77]). The records of *Theridion* species ballooning [40,78] make it highly likely that individuals of our investigated species also balloon while young.

### Sampling

**Fig 1:**
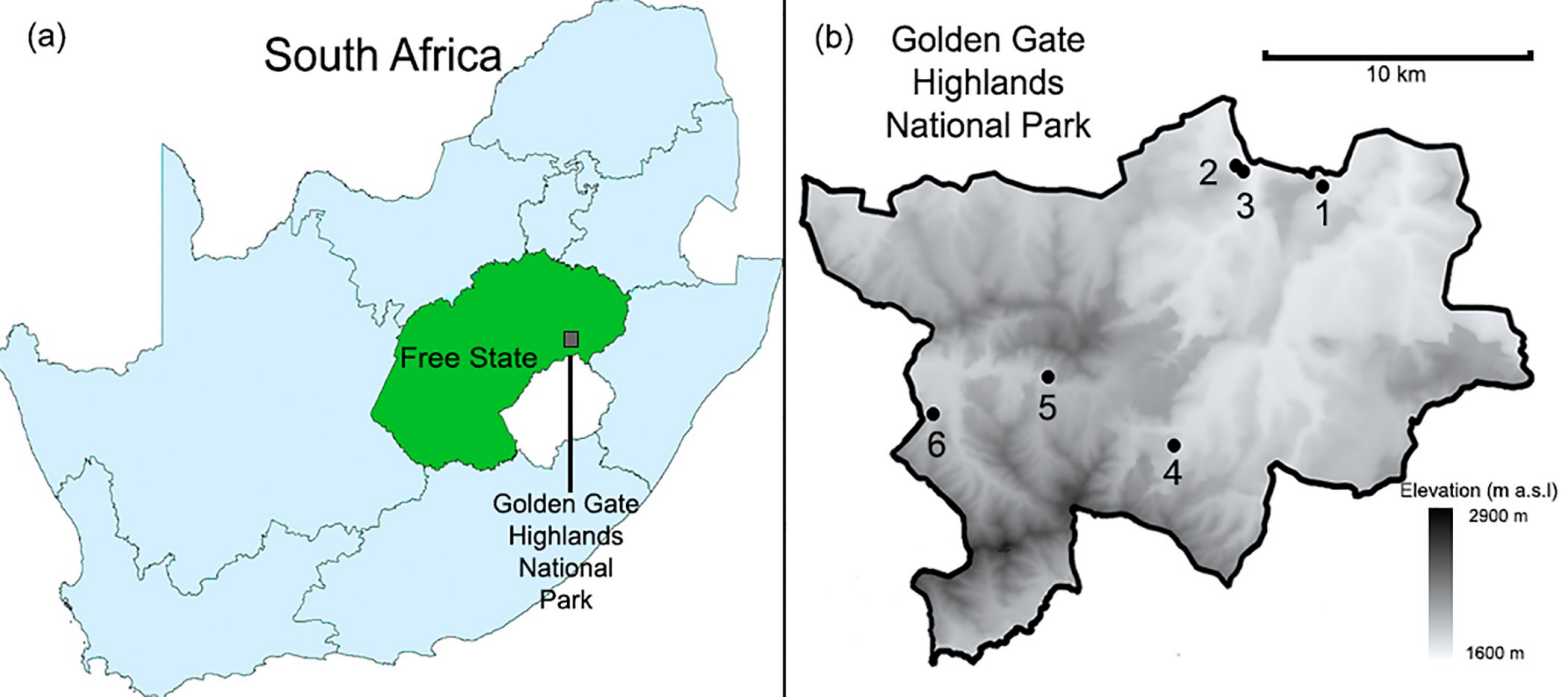
Location of study area. (a) Location of Golden Gate Highlands National Park in the Free State Province, South Africa. (b) Map of the six shrubland sampling localities. Elevation map created using Shuttle Radar Topography Mission (SRTM) terrain height data equal to three seconds of arc latitude and longitude and approximate resolution of 90 m. Developed using QGIS, version 2.18.15. SRTM3 topography data available from USGS Earth Resources Observatory and Science Center (http://eros.usgs.gov/#).

All sampled spider species were collected by beating foliage from tree canopies of six shrubland sites in the GGHNP between April 2018 and March 2019 (Fig 1). Selection of sites was dependant on the prominence of shrublands, as well as ease of accessibility due to financial and time constraints during sampling. Additionally, this is the first study of its kind in the GGHNP, no other studies have been performed in this National Park attempting to investigate fragment barriers and their impact on other species that could be used as a guide in site selection during our study. A maximum of 12 sampled individuals per species per site were intended to be sampled, but certain sites did not yield the desired quantity for certain species (S1, S3, S5, S7 and S9 Tables). All collected specimens (268 in total) were confirmed to belong to the five selected spider species based on morphological characteristics defined from available literature [46]. Of those collected, a total of 249 adult and immature individuals (57 *D*. *purcelli*, 69 *N*. *subfusca*, 34 *P*. *browningi*, 56 *P*. *tuberculatus*, 33 *Theridion* sp.) were successfully utilised in genetic analyses. All specimens were stored in 96% ethyl alcohol under refrigerated conditions until genetic analysis. Collected adult spider specimens were deposited in the National Museum in Bloemfontein (NMBA).

### DNA sequence comparisons

DNA extraction, PCR and Sanger sequencing for each selected individual was performed at the Canadian Centre of DNA Barcoding (CCDB) using standard protocols [79]. One to two legs per individual were removed using sterile forceps, rinsed in 96% ethyl alcohol to limit residual contamination, and transferred into a 96-well microplate pre-filled with approximately 30μl of 96% ethyl alcohol. PCR amplification of the COI-5’ barcode region was conducted using the primer pair C_LepFolF and C_LepFolR [80]. All obtained sequences, collection data, specimen photographs and taxonomic identification of each specimen were deposited on the Barcode of Life Database (BOLD) system under the assigned project “Spiders of South Africa” (SPIZA): *D*. *purcelli* [SPIZA001-19–SPIZA059-19]; *N*. *subfusca* [SPIZA060-19–SPIZA131-19]; *P*. *browningi* [SPIZA132-19–SPIZA174-19]; *P*. *tuberculatus* [SPIZA175-19–SPIZA230-19]; *Theridion* sp. [SPIZA231-19–SPIZA268-19].

For comparative analyses of phylogeny, sequences of our selected species were blasted using the BLASTN algorithm in the NCBI database to identify most homologous species. Voucher sequences of three to six of the most closely related species for each investigated genus were then downloaded (three sequences per species). Due to a lack of voucher sequences of species in the thomisid genus *Pherecydes*, sequences from three species in a closely related genus, namely *Tmarus* Simon, 1875 [81], were obtained. All sequences were aligned per species using MAFFT Multiple sequence alignment version 7 [82–83]. Investigated taxa and voucher sequences were trimmed to a 658bp fragment for all species, with the exception of *P*. *browningi* and its related taxa, which were trimmed to 661bp.

Phylogenetic analyses, for the datasets per species, were performed using Maximum Likelihood (ML) and Bayesian Inference (BI) methods. Model selection for both analyses was performed using the Akaike Information Criterion (AIC, [84]) in jModelTest version 2.1.3 [85]. The ML analyses were conducted in PhyML version 3.1 [86]. Confidence values of branches were assessed via non-parametric bootstrapping with 1000 replicates [87]. Branch supports with bootstrap proportions of 80% or higher were regarded as sufficiently resolved nodes [88–89]. Markov Chain Monte Carlo (MCMC) algorithm was used in the BI, and conducted in MrBayes version 3.2.7 [90]. A total of two million generations were run for each species to ensure appropriate phylogenetic inference, with the exception of *P*. *browningi*, which was run for four million generations due to the increased number of base pairs. Four MCMC chains were run (3 hot, 1 cold), and sampling was performed every 1000 generations, with an adequate burn-in of 10% of the number of generations, determined from likelihood scores and convergence of posterior probabilities [10], for each species. All trees sampled in the burn-in phase were discarded. Topologies with posterior probabilities (PP) of 80% or higher were regarded as sufficiently resolved nodes. In addition, inferred haplotypes and their relationships per sampled species were constructed by the Minimum Spanning Network (MSN) method [91], using PopART, version 1.7 [92].

### Population genetic diversity and gene flow

After alignment and trimming of sequences of the investigated species as described above, the voucher sequences of all related species were removed from the datasets, and only the sequences of our five investigated spider species were used to analyse genetic diversity and gene flow. Investigated taxa sequences were assigned to the six localities from which the specimens were sampled. Levels of *mt*DNA genetic heterogeneity among populations were then quantified according to numbers of segregating sites (S), haplotype number (h), haplotype diversity (Hd), nucleotide diversity (π), and average number of pairwise nucleotide differences within a population (K), as calculated in DnaSP version 6.12.03 [93]. Pairwise genetic differentiation (Fst) between populations were calculated for each investigated species in DnaSP, alongside nucleotide substitution per site (Dxy), while gene flow (Nm) was estimated as mean number of dispersers per generation among the populations.

## Results

### DNA sequence comparisons

#### Dendryphantes purcelli

**Fig 2:**
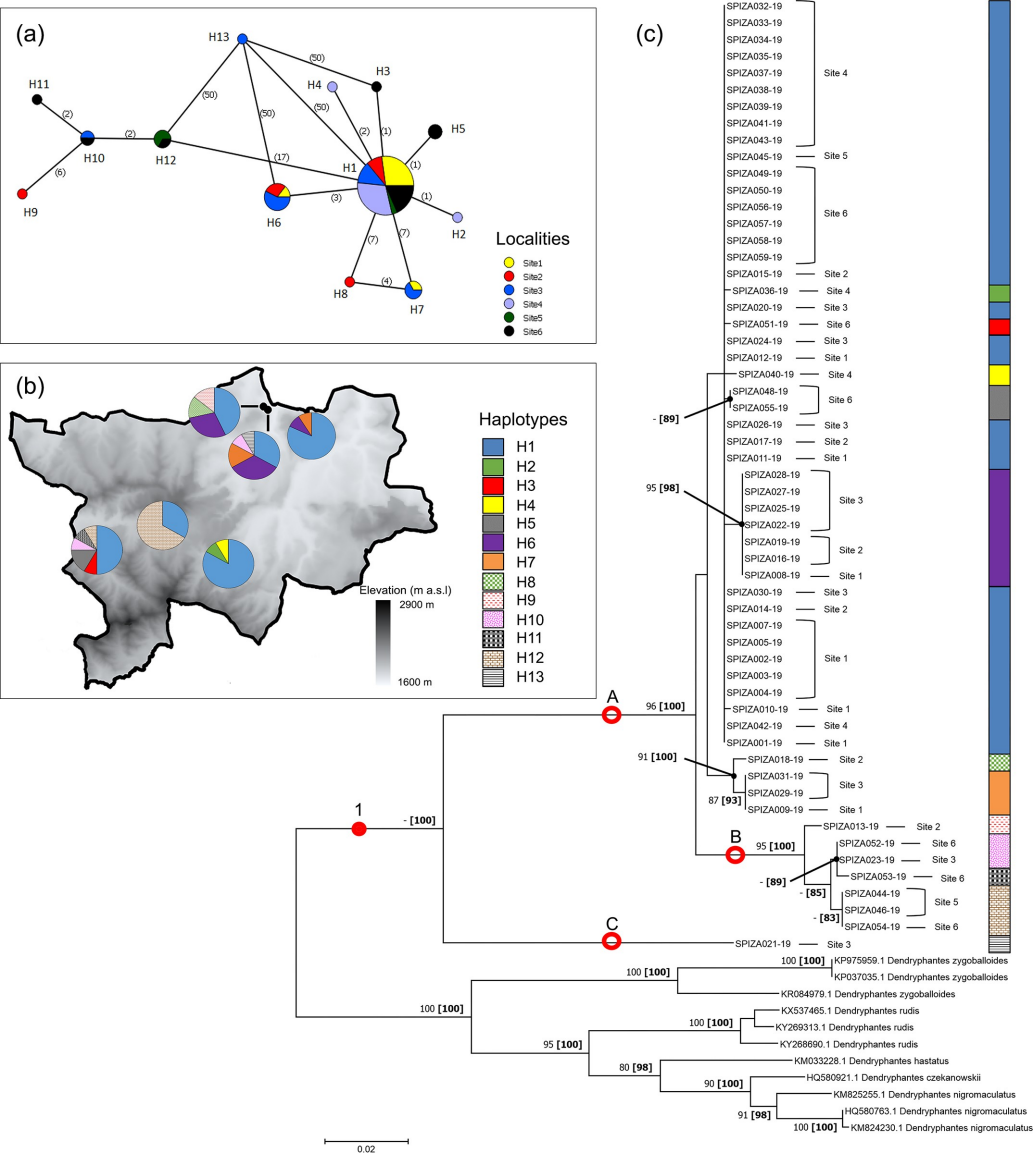
Haplotype networks and phylogram of *Dendryphantes purcelli* populations of the Golden Gate Highlands National Park, based on a portion of *mt*DNA COI gene. (a) Minimum Spanning Network (MSN) connecting sampled sequences through putative mutational steps according to geographical locality of populations. Circles represent defined haplotypes, while circle diameters are proportional to frequency. Numbers in parentheses correspond to mutational steps. (b) Distribution of defined haplotypes within sampled localities. (c) Phylogenetic tree based on Maximum Likelihood (ML) showing relationship of *D*. *purcelli* with five sister species. Red circles and dots represent well-supported and putative clades/groups, respectively. Localities and haplotypes of individuals corresponding to the MSN are also indicated. Phylogram is rooted at midpoint. Numbers at nodes correspond to bootstrap percentages alongside posterior probability percentages in parentheses. Only values >80% were indicated.

A total of 13 haplotypes were identified for *D*. *purcelli* (Fig 2A and 2B). The haplotypes H6 and H7 were shared among the northern populations (Sites 1–3) of the park, and H8 and H9 were only found in Site 2. Among the southern populations (Sites 4–6), haplotypes H2 and H4 only occurred in Site 4, and H3, H5 and H11 only in Site 6. The remaining haplotypes were shared between the remaining localities, with the exception of H13, which was only found in Site 3 and displayed a high number of mutations (50) in comparison to all other haplotypes. Haplotype H1 was the most widespread shared haplotype, occurring in all six localities.

Bayesian and ML phylogeny of the *Dendryphantes* species yielded a tree with strongly supported branches, with the sampled *D*. *purcelli* represented as a single clade (clade 1) by PP (100%), but not bootstrap values, against the other distinctive species (Fig 2C). Sequences of *D*. *purcelli* clustered into two subclades (A and C) that were strongly supported by PP and bootstrap values (100% and 96%, respectively). The first subclade (C) was represented by only one individual, while the second (A) gathered the remaining sequences. Within subclade A, a smaller group (B) was robustly (95% bootstrap, 100% PP) separated from the other sequences. Derived clustering from the park populations showed that collected specimens were independently distributed from their geographic origin. Additionally, all specimens were grouped according to defined haplogroups in the MSN (Fig 2C).

#### Neoscona subfusca

**Fig 3:**
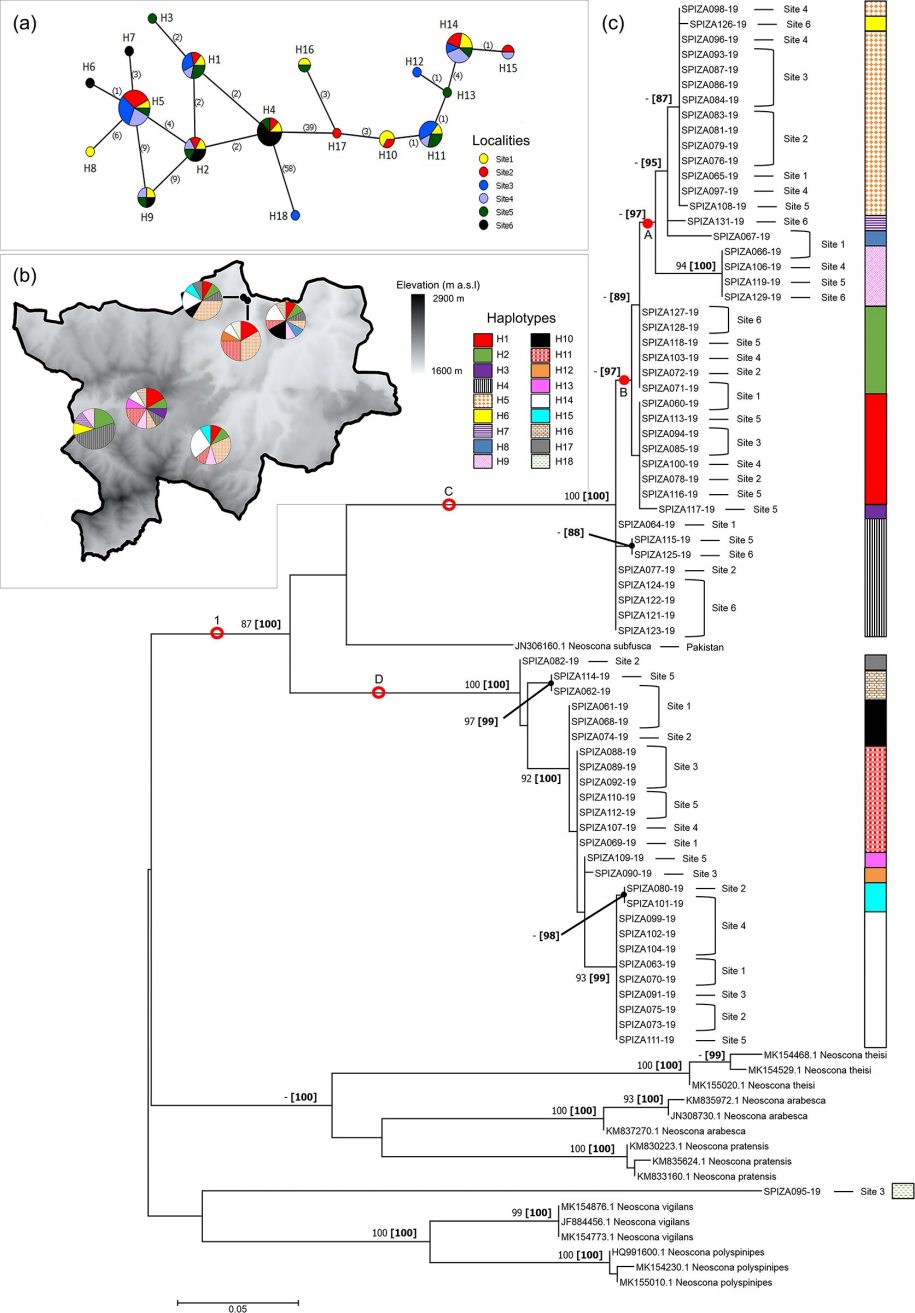
Haplotype networks and phylogram of *Neoscona subfusca* populations of the Golden Gate Highlands National Park, based on a portion of *mt*DNA COI gene. (a) Minimum Spanning Network (MSN) connecting sampled sequences through putative mutational steps according to geographical locality of populations. Circles represent defined haplotypes, while circle diameters are proportional to frequency. Numbers in parentheses correspond to mutational steps. (b) Distribution of defined haplotypes within sampled localities. (c) Phylogenetic tree based on Maximum Likelihood (ML) showing relationship of *N*. *subfusca* with five sister species. Red circles and dots represent well-supported and putative clades/groups, respectively. Localities and haplotypes of individuals corresponding to the MSN are also indicated. Phylogram is rooted at midpoint. Numbers at nodes correspond to bootstrap percentages alongside posterior probability percentages in parentheses. Only values >80% were indicated.

Haplotypes of *N*. *subfusca* totalled 18, with two distinct groupings occurring, separated by a relatively high number of mutations (39) (Fig 3A). The first group comprised haplotypes H1–H9, with H1, H2, H4, H5 and H9 occurring across localities, while H3, H6–H7, and H8 were only found in Sites 5, 6 and 1, respectively. The second group contained haplotypes H10–17, with H12, H13 and H17 occurring in Sites 3, 5 and 2, respectively, while the remainder occurred across the localities except Site 6. Haplotype H18 remained separated from all other haplotypes by a high number of mutations (58) and was only found in Site 3. Haplotypes from both groups were still widely distributed among the investigated localities (Fig 3B).

The two groups of haplotypes constructed by the MSN for *N*. *subfusca* were similarly strongly supported by ML and Bayesian phylogeny (87% and 100%, respectively), with all sequences forming a clade (clade 1) containing two subclades (C and D), with the exception of one individual that grouped close to the voucher sequences of *N*. *vigilans* and *N*. *polyspinipes* (Fig 3C). This individual was therefore considered misidentified as *N*. *subfusca* and belongs to a different species. Each of the two subclades (C and D) contained smaller groupings of sequences that were more strongly supported in subclade D (PP and bootstrap >80%), while subclade C contained more putative groupings (A and B) supported by PP values (>80%) but not bootstrap. The voucher *N*. *subfusca* sequence from Pakistan [94], while not clustering strongly with any of the sequences retrieved from the park, was still seen to group within the clade representing *N*. *subfusca sensu stricto*.

#### Pherecydes tuberculatus

**Fig 4:**
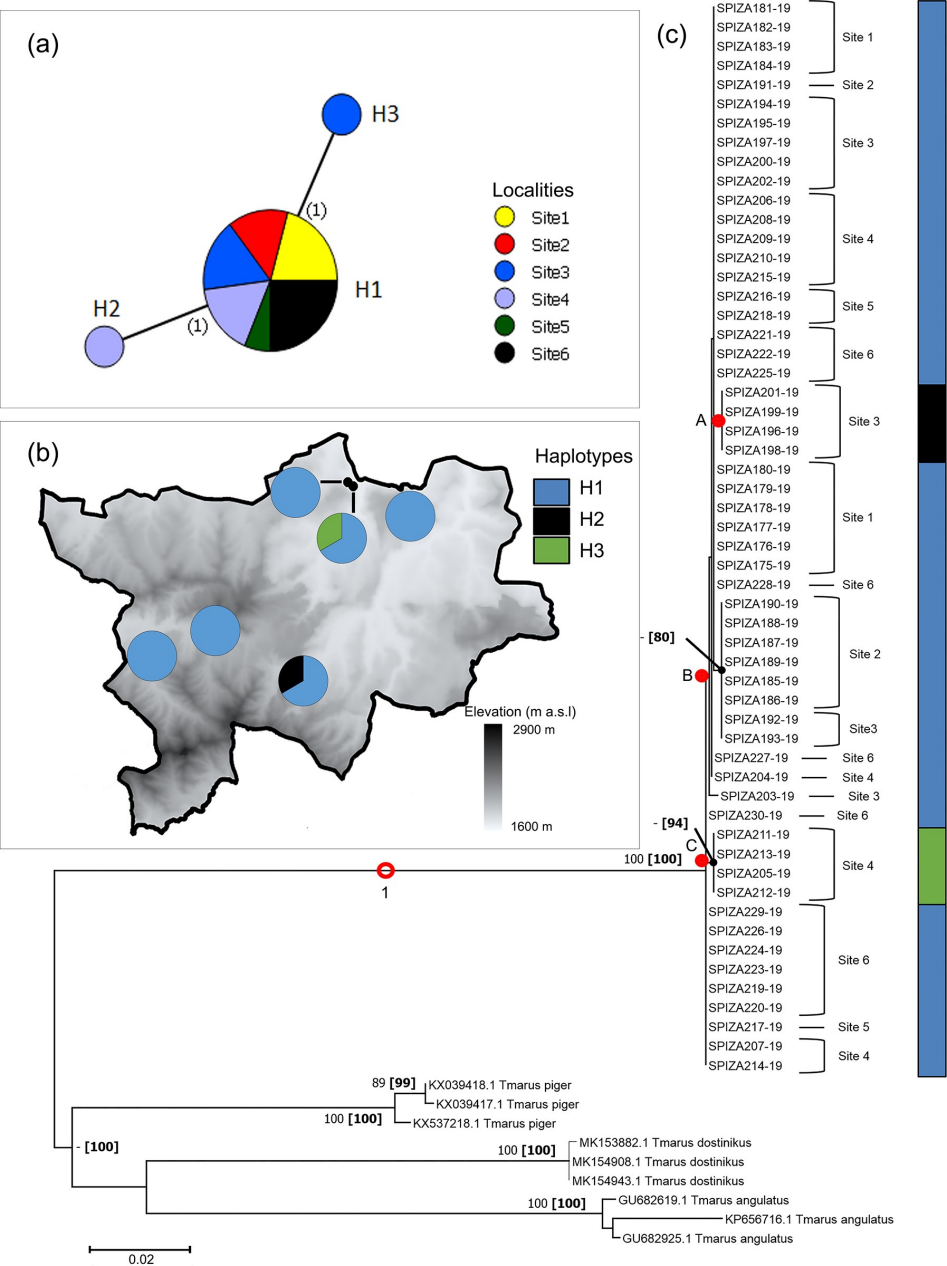
Haplotype networks and phylogram of *Pherecydes tuberculatus* populations of the Golden Gate Highlands National Park, based on a portion of *mt*DNA COI gene. (a) Minimum Spanning Network (MSN) connecting sampled sequences through putative mutational steps according to geographical locality of populations. Circles represent defined haplotypes, while circle diameters are proportional to frequency. Numbers in parentheses correspond to mutational steps. (b) Distribution of defined haplotypes within sampled localities. (c) Phylogenetic tree based on Maximum Likelihood (ML) showing relationship of *P*. *tuberculatus* with three species from a related genus. Red circles and dots represent well-supported and putative clades/groups, respectively. Localities and haplotypes of individuals corresponding to the MSN are also indicated. Phylogram is rooted at midpoint. Numbers at nodes correspond to bootstrap percentages alongside posterior probability percentages in parentheses. Only values >80% were indicated.

Only three haplotypes were identified for *P*. *tuberculatus*, with only a single mutation occurring between them (Fig 4A). Haplotypes H2 and H3 only occurred in Sites 4 and 3, respectively, while H1 was shared among all six populations (Fig 4B).

All obtained sequences of *P*. *tuberculatus* formed a strongly supported species (100% bootstrap, 100% PP), clearly separate from the *Tmarus* species included in the analyses (Fig 4C). Within the *Pherecydes* clade (clade 1), a single subclade (B), as well as numerous groupings of specimens were seen. All *P*. *tuberculatus* sequences in the phylogram were found to belong to haplotype H1, with the exception of two heterogeneous groups (A and C), one of which (C) was strongly supported by PP (94%), but not bootstrap values.

#### Philodromus browning

**Fig 5:**
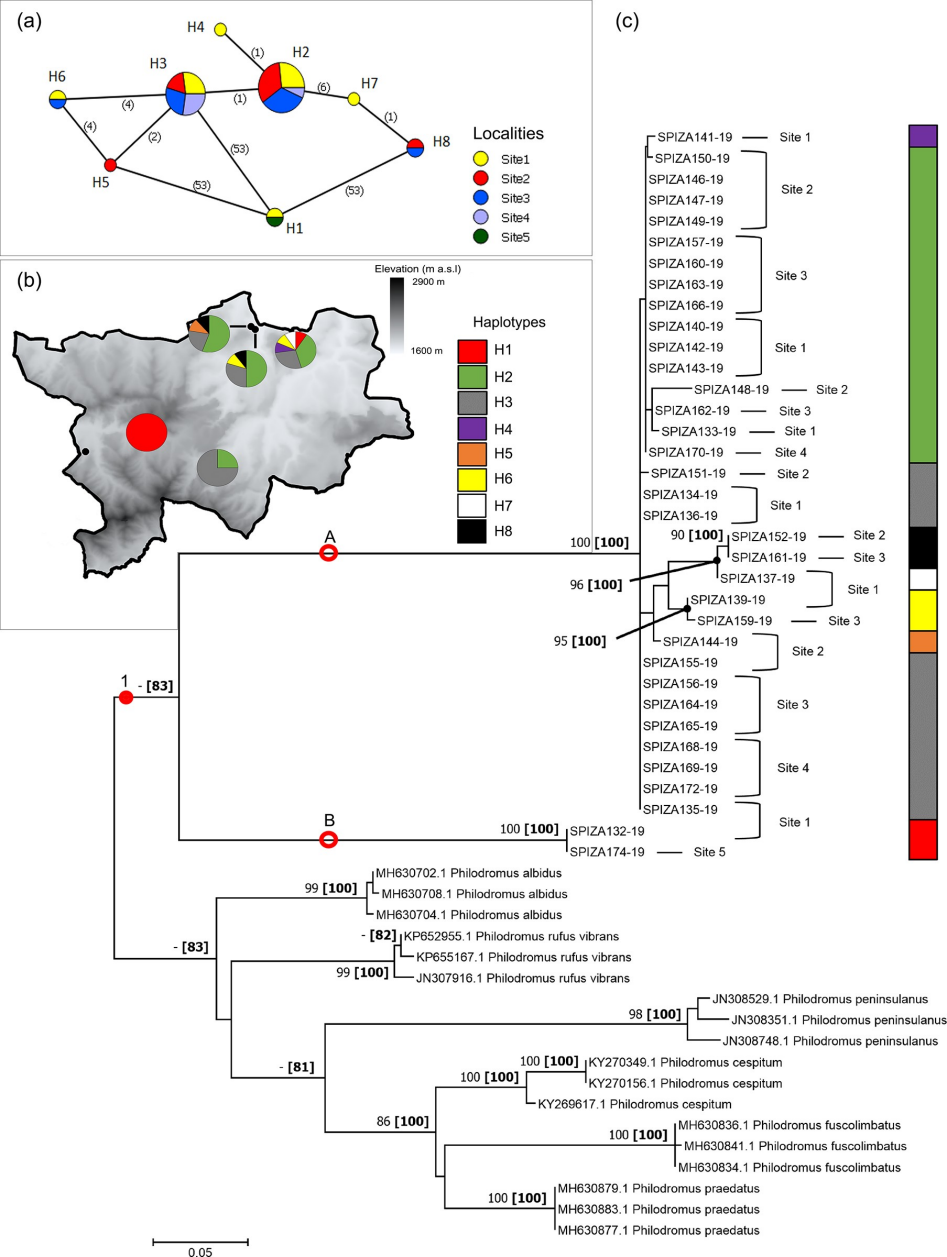
Haplotype networks and phylogram of *Philodromus browningi* populations of the Golden Gate Highlands National Park, based on a portion of *mt*DNA COI gene. (a) Minimum Spanning Network (MSN) connecting sampled sequences through putative mutational steps according to geographical locality of populations. Circles represent defined haplotypes, while circle diameters are proportional to frequency. Numbers in parentheses correspond to mutational steps. (b) Distribution of defined haplotypes within sampled localities. (c) Phylogenetic tree based on Maximum Likelihood (ML) showing relationship of *P*. *browningi* with six sister species. Red circles and dots represent well-supported and putative clades/groups, respectively. Localities and haplotypes of individuals corresponding to the MSN are also indicated. Phylogram is rooted at midpoint. Numbers at nodes correspond to bootstrap percentages alongside posterior probability percentages in parentheses. Only values >80% were indicated.

The majority of the eight haplotypes of *P*. *browningi* were shared across most of the localities, with the exception of H4 and H7, which were only found in Site 1, and H5 that was only found in Site 2 (Fig 5A). Haplotype H1 separated from the other haplogroups, due to a high number of mutations (53), and occurred in Sites 1 and 5. The haplotypes H2 and H3 were the most commonly shared haplotypes, occurring in Sites 1–4, while H8 was only found in Sites 2 and 3 (Fig 5B).

Sampled sequences of *P*. *browningi* formed a clade (clade 1) that was strongly supported by PP (83%), but not bootstrap values (Fig 5C). These sequences formed two subclades (A and B), with subclade B containing only two individuals, and subclade A including the remainder. Strongly supported groupings (>90% for both bootstrap and PP) of specimens were noted in subclade A according to defined haplogroups. Due to weakly supported branches, certain specimens, initially considered to be different haplogroups, were indicated as belonging to other haplotypes (Fig 5C).

#### *Theridion* sp

**Fig 6:**
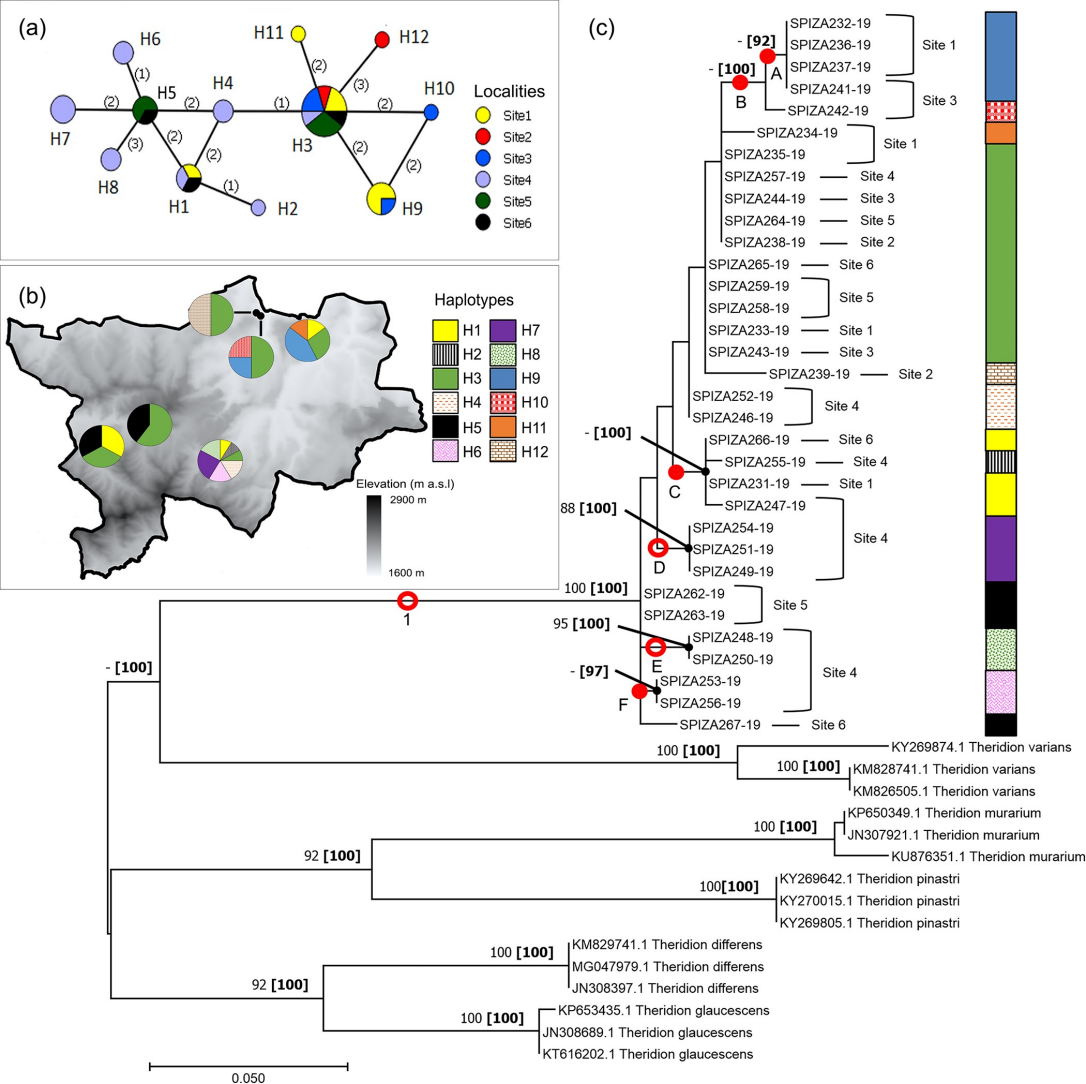
Haplotype networks and phylogram of the *Theridion* sp. populations of the Golden Gate Highlands National Park, based on a portion of *mt*DNA COI gene. (a) Minimum Spanning Network (MSN) connecting sampled sequences through putative mutational steps according to geographical locality of populations. Circles represent defined haplotypes, while circle diameters are proportional to frequency. Numbers in parentheses correspond to mutational steps. (b) Distribution of defined haplotypes within sampled localities. (c) Phylogenetic tree based on Maximum Likelihood (ML) showing relationship of the *Theridion* sp. with five sister species. Red circles and dots represent well-supported and putative clades/groups, respectively. Localities and haplotypes of individuals corresponding to the MSN are also indicated. Phylogram is rooted at midpoint. Numbers at nodes correspond to bootstrap percentages alongside posterior probability percentages in parentheses. Only values >80% were indicated.

A total of 12 haplotypes were identified for the *Theridion* sp. investigated (Fig 6A). Haplotype H3 was the most commonly shared haplotype, occurring in all populations (Fig 6B). Haplotypes H9–H12 were exclusive to the northern populations of the park, with H10, H11 and H12 only occurring in Site 3, 1 and 2, respectively, while H9 was shared between Sites 1 and 3. Populations on the south-western side of the park were seen to share haplotype H5, while H1 was shared between Sites 1, 4 and 6. All other haplotypes were only found in Site 4.

A strongly supported monophyletic clade (clade 1) of sampled *Theridion* sequences was represented by Bayesian and ML phylogenies (100% for both) (Fig 6C). Within this single clade a number of groups (A–F) occurred, two of which (D and E) were strongly supported by PP and bootstrap values (>80%). The remaining four groups were strongly supported by PP values (>80%) but not bootstrap. Clustering from populations in the park indicated independent distribution of specimens from their geographic origin. Haplogroups defined by the MSN were represented by grouping of sequences in the phylogeny.

### Population genetic diversity and gene flow

As a single individual of the sampled *N*. *subfusca* specimens (SPIZA095-19) was considered to be a different species based on the obtained phylogeny (Fig 3C), this individual was removed from the dataset before genetic diversity and gene flow were calculated. Nevertheless, *N*. *subfusca* was observed to possess the most haplotypes (17 as opposed to the original 18 defined in the MSN and phylogeny), supporting a high haplotype (Hd = 0.9078) and nucleotide diversity (π = 0.0413) (Table 1). While the phylogenies of *D*. *purcelli* and *P*. *browningi* were also noted to contain individuals (*D*. *purcelli*–SPIZA021-19; *P*. *browningi*–SPIZA132-19 and SPIZA174-19) that may be interpreted as different species, these individuals were still represented in the major clade of their respective species (*D*. *purcelli*–Fig 2C; *P*. *browningi*–Fig 5C). In this regard, these individuals were retained during genetic diversity and gene flow calculations. As such, *D*. *purcelli* was seen to have the second highest number of haplotypes (h = 13), but did not possess the second highest haplotype diversity (Hd = 0.6510), which instead occurred in the *Theridion* sp. (Hd = 0.8807) (Table 1). Lowest haplotype diversity was seen in *P*. *tuberculatus* (Hd = 0.2597), with only three haplotypes being found across the six populations.

**Table 1:** Total diversity indices of the five investigated spider species from populations in the Golden Gate Highlands National Park, calculated from nucleotide sequence of mitochondrial COI gene.

Species	*Dendryphantes purcelli*	*Neoscona subfusca*	*Pherecydes tuberculatus*	*Philodromus browningi*	*Theridion* sp.
N	57	68	56	34	33
S	71	64	2	59	18
h	13	17	3	8	12
Hd	0.6510	0.9078	0.2597	0.7112	0.8807
Kt	7.4317	23.9723	0.2701	5.2513	3.1288
πT	0.0135	0.0413	0.0005	0.0136	0.0064
Fst	0.1503	0.0424	0.2727	0	0.1354
Nm	1.41	5.65	0.67	0	1.60

N: Number of sequences; S: Number of segregating (polymorphic/variable) sites; h: Number of haplotypes; Hd: Total haplotype diversity; Kt: Average number of nucleotide differences; πT: Total nucleotide diversity; Fst: Overall genetic differentiation; Nm: Gene flow.

Gene flow estimation was high among the populations of *D*. *purcelli* (Nm = 1.41), *N*. *subfusca* (Nm = 5.65) and the *Theridion* sp. (Nm = 1.60), while *P*. *tuberculatus* and *P*. *browningi* showed low levels of connectivity (Nm < 1.0) (Table 1). Genetic differentiation (Fst) mirrored these results, as those species with a high gene flow estimation were seen to possess a moderate to low genetic divergence (Fst < 0.16), while those with a lower rate of gene flow saw a higher divergence (Fst > 0.25) occurring among the populations. Unexpectedly, the gene flow of *P*. *browningi* was estimated to be zero, based on the current samples, with no genetic differentiation across the populations (Fst = 0). However, due to the lack of sufficient specimens from two of the sampled localities (Sites 5 and 6) this result remains debatable.

The populations of *D*. *purcelli* indicated the highest haplotype diversity to occur in Site 2 (Hd = 0.8095), while the highest nucleotide diversity and differences occurred in Site 3 (π = 0.0260, K = 14.3333) (S1 Table). Additionally, the population of Site 6 contained the highest number of haplotypes (h = 6), while Site 5 contained the lowest (h = 2). In terms of genetic differentiation, Site 5 was seen to possess large divergence values (Fst > 0.15) compared to all other sites, with the exception of Site 6 (Fst = 0.0767) (S2 Table). It was also noted that a moderate genetic divergence (Fst = 0.1181) occurred between the two farthest populations of Site 1 and 6. Overall, genetic variability showed less genetic divergence (Fst < 0.1) occurring among the northern (Site 1–3) and eastern (Site 4) populations of the park. Average number of nucleotide substitutions per site between all the populations (Dxy) varied from 0.0095 (Site 4 and 6) to 0.0285 (Site 3 and 5) (S2 Table).

Haplotype diversity was relatively high across all populations of *N*. *subfusca* (Hd > 0.75), with the highest diversity shared between Sites 1 and 5 (Hd = 0.9697) (S3 Table). These two populations concurrently contained the highest number of haplotypes (h = 10). Additionally, the highest nucleotide diversity and differences was seen in Site 1 (π = 0.0473, K = 27.4546). Genetic differentiation showed no genetic divergence (Fst = 0) occurring between the populations, with the exception of Site 6, in which very large divergences (Fst > 0.25) were noted against all other populations (S4 Table). Dxy values between the populations were also noted to be relatively similar (0.0354–0.0437).

Due to only a single haplotype occurring across all populations of *P*. *tuberculatus*, with the exception of Sites 3 and 4, haplotype and nucleotide diversity was indicated as zero (S5 Table). Sites 3 and 4, on the other hand, each possessed an additional unique haplotype per locality, increasing diversity of these two populations (Hd = 0.4849, π = 0.0010). As only a single haplotype occurred in the majority of populations, genetic divergence was non-existent between Sites 1, 2, 5 and 6, as both Fst and Dxy values were zero (S6 Table), indicating no genetic variability between these populations. However, the same very large Fst value (0.2727) was noted for pairwise comparisons of Sites 3 and 4 against all other populations, due to these two sites possessing their own unique additional haplotype. Furthermore, average number of nucleotide substitutions per site between Sites 3 and 4 and the remaining populations were identical (Dxy = 0.0007), while the pairwise comparison between Sites 3 and 4 portrayed a higher value (Dxy = 0.0013) (S6 Table).

Despite a lack of sufficient specimens in Sites 5 and 6, the remaining populations of *P*. *browningi* indicated Site 1 as possessing the highest haplotype and nucleotide diversity (Hd = 0.8364, π = 0.0310), while Site 4 possessed the lowest (Hd = 0.5000, π = 0.0013) (S7 Table). Estimates of genetic differentiation showed no genetic divergence (Fst = 0) occurring between the northern populations (Sites 1–3), while moderate divergence (Fst average = 0.0458) was experienced between the eastern population (Site 4) and those in the north (S8 Table). Dxy values were also noted to differ between the populations, ranging from a minimum of 0.0060 between Sites 2 and 3 to a maximum of 0.0183 between Sites 1 and 3. However, further sampling is necessary from populations of Sites 5 and 6 to properly gauge genetic differentiation and gene flow across the park.

While the *Theridion* sp. saw a maximum haplotype diversity occurring in Sites 2 and 6 (Hd = 1) (S9 Table), the fact that the number of individuals sampled was low (N < 4), and the number of haplotypes identified in these localities was equivalent to their sample sizes (Site 2, h = 2; Site 6, h = 3), may not represent true haplotype diversity in these populations. As such, the haplotype diversity indicated in Site 4 (Hd = 0.9091) is instead regarded as the population with the highest diversity, due to a larger sample size (N = 12), with Site 5 possessing the lowest diversity (Hd = 0.6000). Nucleotide diversity was also noted to follow the same trend (Site 4, π = 0.0074), however, lowest nucleotide diversity was instead seen in Site 3 (π = 0.0034) (S9 Table). Genetic differentiation was observed to be relatively low (Fst < 0.1) among the majority of the localities, with Sites 1 with 3, and 6 with 4 and 5, indicating a lack of barriers to gene flow (Fst = 0) (S10 Table). Site 4 was also noted to possess a very large genetic divergence (Fst > 0.25) with the northern populations (Sites 1–3). Average number of nucleotide substitutions per site were noted to follow the trends seen in the Fst values, with the highest number of substitutions occurring between Sites 2 and 4 (Dxy = 0.0106).

## Discussion

This was the first study conducted in a South African National Park that attempted to determine the state of gene flow of five common spider species, and their inferred dispersal capability, between selected shrubland populations. The results indicate that the majority of the investigated species were able to maintain gene flow between the selected populations despite the mountainous terrain. This occurrence was unexpected but not unwarranted, as previous cases of gene flow in spiders have noted smaller mountain ranges to not be a major geographic barrier to certain species [22,95]. Consequently, genetic heterogeneity was relatively high among the selected populations of the investigated taxa, as quantified by haplotype diversity, with the exception of *P*. *tuberculatus*. It was also observed that the two web-building species, *N*. *subfusca* and *Theridion* sp., possessed the highest number of dispersers per generation, along with the lowest overall genetic divergence, of the five investigated taxa.

Subsequent analyses based on the COI gene indicated that the majority of the investigated spider species harbour high haplotype diversity among the sampled shrubland localities of the GGHNP, alongside moderate to low nucleotide diversity. Similar occurrences were reported in analyses of spider species from other countries [10,96–97], albeit on a larger geographical scale. Unfortunately, few reports on the genetic diversity of South African araneid populations are available for comparison with our study (e.g. [98]). While genetic diversity was relatively high among our investigated taxa, genetic differentiation was moderate to low, implying the possibility of such diversity being maintained by gene flow [10]. Additionally, the current reported genetic variability experienced by the populations of the five investigated taxa may be the result of unequal gene flow transfer between the selected localities [99–100].

A lower rate of gene flow was observed among the *P*. *tuberculatus* and *P*. *browningi* populations, where less than one individual per generation was estimated. Such decreased connectivity was exemplified by their genetic diversity, with the exception of *P*. *browningi* that indicated a relatively high diversity despite a total of zero dispersers being estimated. Decreased gene flow has been reported for members of Thomisidae and Philodromidae [101–103], where substantial inbreeding within locales was observed and postulated. These occurrences have even been reported at relatively small scales (~100 m x 100 m) [104]. Such poor dispersal abilities may lead to a build-up of genetic structure within populations, decreasing genetic heterogeneity over a period of generations, particularly among social spiders [105–106]. Additionally, the Drakensberg itself is relatively poorly represented in collections by identified spider specimens, with neither of these two species being previously recorded from localities along the mountain range [52]. This indicates a sizeable gap in knowledge regarding current population ranges, as it is uncertain whether these species regularly occur along the mountainous areas and whether this may have impacted gene flow and genetic diversity in the past. Furthermore, the sample sizes of these two species obtained during this study must also be considered (S5 and S7 Tables), as they may not have provided proper representation of genetic diversity and gene flow within the GGHNP.

Overall, higher genetic differentiation was observed to occur more commonly between the northern and western locales of the park for the majority of the investigated taxa. This was especially evident for *D*. *purcelli*, *N*. *subfusca* and *P*. *browningi*. Locales in the western section were more elevated than those in the north, and differences in the dominant vegetation between these localities were observed (S11 Table). Habitat structure and fragmentation are well known to influence genetic exchange between populations, depending on species’ behaviour and dispersal capabilities [107–110]. In certain instances, an agricultural area of 100 m can present a complete barrier to gene flow for certain spider species, due to removal of natural vegetation [111]. Some spider species are also known to prefer certain types of vegetation over others [60,112–115], which may impact their ability to colonise new habitats. Such preference is often documented in orb-weaver species, such as *N*. *subfusca*, in which vegetation complexity often determines site selection [116–118]. Additionally, basic movement of air masses across the park occur south-easterly during the summer and winter seasons, with certain degrees of largescale anticlockwise circulation across the entire eastern Free State [119]. This general wind direction further supports the results obtained regarding genetic differentiation, as the south-easterly winds may limit dispersal of individuals from the northern localities (Sites 1, 2 and 3) to the western (Sites 5 and 6) and eastern (Site 4) sites, while still maintaining connectivity among the northern localities. Furthermore, when examining the topography of the park a distinct elevation range occurs across the north-western section of the park, extending further west and south, with a number of gulleys and depressions present. However, it is still unclear whether this elevated area may represent a large orographic barrier within the confines of the park, and to what extent it could be a major obstacle to the aerial and terrestrial dispersal of spider species [10]. Further studies investigating gene flow in localities across the full elevational and geographical range of the park must therefore be considered.

Obtained results indicated that the two web-building species, *N*. *subfusca* and *Theridion* sp., possessed the highest rates of gene flow among the investigated taxa. Wind-mediated dispersal of spiders via ballooning is frequently observed globally [120–121]. While ballooning is widely considered to be the primary mechanism of habitat colonisation by spiders [39,64,122], its role in maintaining genetic heterogeneity between populations has been considered to be more prevalent in web-building spider populations [123–125]. Orb-web spider species may maintain genetic exchange over a distance of 23.6 km across a fragmented landscape [6,126]. However, it was also speculated by Ramirez and Haakonsen [6] that populations of araneids situated in a matrix of primarily unfavourable habitat may have little difficulty in maintaining genetic connectedness. These authors also noted that ballooning may not confer unlimited access among all populations in an area, suggesting this type of aerial dispersal may be less effective over long distances.

Opinion as to whether ballooning is an obligatory life history phase has also remained polarised [39,123,127], with certain reports indicating it as non-essential for certain species [128]. This brings into question the use of ballooning in the five spider species we investigated. Therefore, while the high gene flow and increased connectivity between the majority of populations of *N*. *subfusca* and the *Theridion* sp. may be explained, at least in part, by the possibility of ballooning in these species, proper investigation as to the degree and commonality of this mechanism in South African spider species is still needed.

From the results obtained it can be considered that at least some dispersal and gene flow was occurring between all the populations sampled during this study, with certain populations experiencing fewer to no dispersal barriers, depending on distance and location. Such occurrences may allow for the amelioration of deleterious effects of inbreeding for the majority of the investigated populations [129]. As theorised by Wade and McCauley [130], a good dispersal model is implied by high Nm values within populations that are associated with high levels of genetic diversity, given that random samples are recruited from previous living populations, or vice versa, across a geographic region. It could therefore be considered from our results that, out of the five investigated species, *N*. *subfusca* possesses a good dispersal model within the confines of the park, due to the high genetic diversity and dispersal rate seen among the selected populations. However, the GGHNP can be regarded as only a small part of the larger shrubland-containing topography of the Drakensberg mountain range [131]. This has implications when attempting to sufficiently determine the genetic diversity and dispersal ability of these spider species, as other relevantly comparable populations on a larger geographical scale become excluded from analyses. Additionally, the six populations of each species investigated during our study only represent a subset of their overall populations in the park. As such, further studies are needed to determine the demographic history and full population expansion of all five investigated species not only in the GGHNP itself, but across the larger Drakensberg range.

Another point of consideration regarding gene flow results of these spider species is the implication of uniparental inheritance and sex-biased dispersal. Due to only *mt*DNA being utilised in this study, there is the possibility that some of the haplotypes detected may have been from male lineages no longer existing, inflating the contemporary diversity seen in this study [132]. Mated adult female spiders are often responsible for gene flow, with little or no male gene flow among populations, resulting in highly inbred populations and large genetic differentiation between localities at both maternal and biparental inherited loci [133–134]. However, according to Greenstone et al. [36] the dominant mode of dispersal in many spider species is pre-mating dispersal of juveniles, which results in an out-breeding mating system. Additionally, sub-social spider species have been seen to exhibit male and female gene flow after the group phase, although a general reduction in gene flow for both sexes leads to sub-structured populations where the females are genetically related and this population is governed by female group-founding [135–138]. The inclusion of additional microsatellites that are not subject to the evolutionary constraints of *mt*DNA would provide a clearer picture of the state of gene flow of these spider species in the GGHNP and beyond.

Incidentally, certain phylogenies indicated the possibility of more than one species among the taxa investigated here. Particularly, populations of *D*. *purcelli*, *N*. *subfusca* and *P*. *browningi* were highly heterogeneous in the GGHNP. Although *D*. *purcelli* and *P*. *browningi* comprised subclades that, while containing only a few individuals, were strongly supported, the sampled individuals of *N*. *subfusca* were unequally divided into two distinct subclades. As individuals of these three species were distributed in the same geographical area (the GGHNP), these clades may be considered as sympatric [97]. Additionally, due to the strength of the bootstrap and PP values of the two subclades of *N*. *subfusca* it can be reliably concluded that they are different species. However, while all sampled individuals were identified according to current morphological characteristics and descriptions, the reliability of identifications may have been detracted due to some of the sampled specimens being juveniles, as juveniles often cannot be reliably identified to species level for certain species, contributing to a possible source of error. Nevertheless, the observation of such heterogeneity over a relatively small geographical area not only brings into question the extent of species complexes, and the subsequent implications for conservation, but also highlights issues regarding the reliability and extent of current taxonomic descriptions and identification of these species in South Africa.

In conclusion, molecular analyses were performed to identify the current state of gene flow and inferred dispersal capability of five ubiquitous spider species from six shrubland localities of the GGHNP. We found that the majority of the investigated species were able to maintain a high rate of gene flow despite the mountainous landscape. We also observed that all species, except *P*. *tuberculatus*, possessed relatively high genetic diversity, together with moderate to low genetic differentiation, which may be the result of overall maintained gene flow across the sampled sites. The inference of ballooning and its role in gene flow of the five investigated species is still under debate, due to a lack of information from South African studies. However, the two-web building species (*N*. *subfusca* and *Theridion* sp.) possessed higher rates of gene flow and less genetic differentiation compared to the other species. Unexpectedly, instances of high heterogeneity in phylogenies of *D*. *purcelli*, *N*. *subfusca* and *P*. *browningi* bring into question the extent of species complexes present in these taxa, concerning current taxonomic descriptions and identification. Due to such genetic diversity and variability observed over a relatively small geographical range, further analyses using additional molecular markers and extended sampling are required to: (i) fully gauge the extent of genetic heterogeneity, gene flow and dispersal ability of spider species within the GGHNP and beyond in the Drakensberg Mountains, and (ii) identify factors that may affect and promote diversity in these selected spider species.

## Supporting information

S1 Fig Dorsal habitus of the five investigated spider species sampled from the Golden Gate Highlands National Park. *Dendryphantes purcelli* Peckham & Peckham, 1903 female and male (a and b), *Neoscona subfusca* (C.L. Koch, 1837) female and male (c and d), *Pherecydes tuberculatus* O.P.-Cambridge, 1883 female and male (e and f), *Philodromus browningi* Lawrence, 1952 female and male (g and h), and *Theridion* sp. females, showing colour variation (i and j).
(TIF)

S1 Table Diversity indices of *Dendryphantes purcelli* populations in the Golden Gate Highlands National Park, calculated from nucleotide sequence of the mitochondrial COI gene. (PDF)

S2 Table Pairwise genetic differentiation (Fst) and nucleotide substitution per site (Dxy) among Golden Gate Highlands National Park populations of *Dendryphantes purcelli*. Fst values are represented in the bottom triangle of the matrix and Dxy values are represented in the top.
(PDF)

S3 Table Diversity indices of *Neoscona subfusca* populations in the Golden Gate Highlands National Park, calculated from nucleotide sequence of the mitochondrial COI gene. (PDF)

S4 Table Pairwise genetic differentiation (Fst) and nucleotide substitution per site (Dxy) among Golden Gate Highlands National Park populations of *Neoscona subfusca*. Fst values are represented in the bottom triangle of the matrix and Dxy values are represented in the top.
(PDF)

S5 Table Diversity indices of *Pherecydes tuberculatus* populations in the Golden Gate Highlands National Park, calculated from nucleotide sequence of the mitochondrial COI gene. (PDF)

S6 Table Pairwise genetic differentiation (Fst) and nucleotide substitution per site (Dxy) among Golden Gate Highlands National Park populations of *Pherecydes tuberculatus*. Fst values are represented in the bottom triangle of the matrix and Dxy values are represented in the top.
(PDF)

S7 Table Diversity indices of *Philodromus browningi* populations in the Golden Gate Highlands National Park, calculated from nucleotide sequence of the mitochondrial COI gene. (PDF)

S8 Table Pairwise genetic differentiation (Fst) and nucleotide substitution per site (Dxy) among Golden Gate Highlands National Park populations of *Philodromus browningi*. Fst values are represented in the bottom triangle of the matrix and Dxy values are represented in the top.
(PDF)

S9 Table Diversity indices of the *Theridion* sp. populations in the Golden Gate Highlands National Park, calculated from nucleotide sequence of the mitochondrial COI gene. (PDF)

S10 Table Pairwise genetic differentiation (Fst) and nucleotide substitution per site (Dxy) among Golden Gate Highlands National Park populations of the *Theridion* sp. Fst values are represented in the bottom triangle of the matrix and Dxy values are represented in the top.
(PDF)

S11 Table Table indicating the dominant plant species present at the time of sampling in each site. (PDF)
