# Supplementary figures and images for: High genetic diversity of spider species in a mosaic montane grassland landscape
Source: PLoS One. 2020 Jun 8;15(6):e0234437. doi: 10.1371/journal.pone.0234437 (PMC7279597; doi:10.1371/journal.pone.0234437)

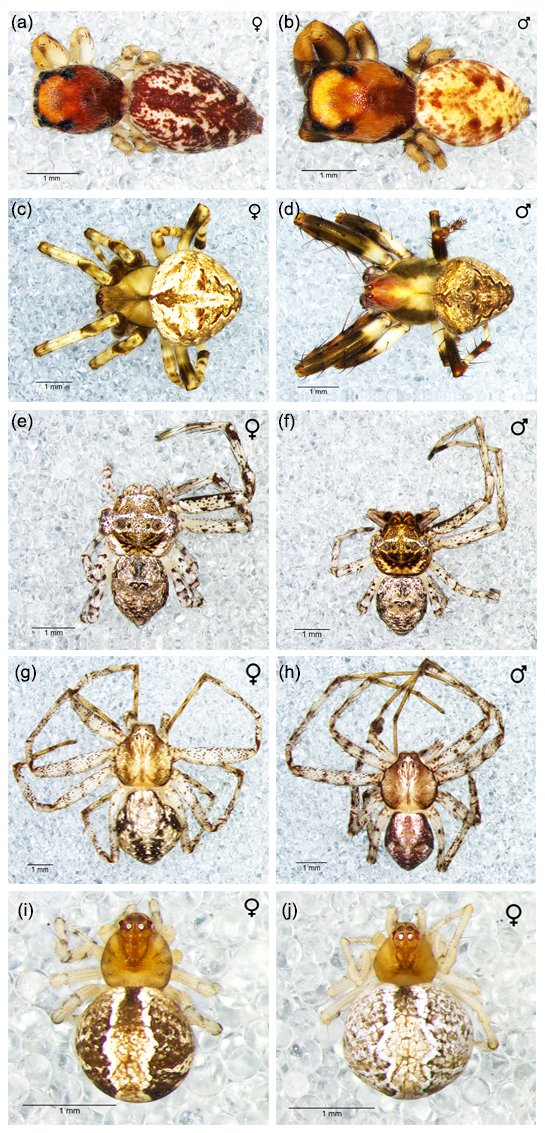

Supplement: S1 Fig — Dendryphantes purcelli Peckham & Peckham, 1903 female and male (a and b), Neoscona subfusca (C.L. Koch, 1837) female and male (c and d), Pherecydes tuberculatus O.P.-Cambridge, 1883 female and male (e and f), Philodromus browningi Lawrence, 1952 female and male (g and h), and Theridion sp. females, showing colour variation (i and j). (TIF) [file pone.0234437.s001.tif]
