# Supplementary material for: High genetic diversity of spider species in a mosaic montane grassland landscape
Source: PLoS One. 2020 Jun 8;15(6):e0234437. doi: 10.1371/journal.pone.0234437 (PMC7279597; doi:10.1371/journal.pone.0234437)
Supplement: S1 Table — (PDF) [file pone.0234437.s002.pdf]

S1 Table. Diversity indices of *Dendryphantes purcelli* populations in the Golden Gate Highlands National Park, calculated from nucleotide sequence of the mitochondrial COI gene

Site	N	S	h	Hd	K	π
Site 1	11	10	3	0.3455	1.8182	0.0033
Site 2	7	25	4	0.8095	8.3810	0.0152
Site 3	12	64	5	0.8030	14.3333	0.0260
Site 4	12	3	3	0.3182	0.5000	0.0009
Site 5	3	17	2	0.6667	11.3333	0.0205
Site 6	12	23	6	0.7576	8.3636	0.0152
Total	57	71	13	0.6510	7.4317	0.0135

N: Number of sequences; S: Number of segregating (polymorphic/variable) sites; h: Number of haplotypes; Hd: Haplotype diversity; K: Average number of nucleotide differences; π: Nucleotide diversity.
