# Supplementary material for: High genetic diversity of spider species in a mosaic montane grassland landscape
Source: PLoS One. 2020 Jun 8;15(6):e0234437. doi: 10.1371/journal.pone.0234437 (PMC7279597; doi:10.1371/journal.pone.0234437)
Supplement: S2 Table — Fst values are represented in the bottom triangle of the matrix and Dxy values are represented in the top. (PDF) [file pone.0234437.s003.pdf]

S2 Table. Pairwise genetic differentiation (Fst) and nucleotide substitution per site (Dxy) among Golden Gate Highlands National Park populations of *Dendryphantes purcelli*. Fst values are represented in the bottom triangle of the matrix and Dxy values are represented in the top.

	1	2	3	4	5	6
1		0.0092	0.0151	0.0021	0.0215	0.0105
2	0		0.0194	0.0087	0.0230	0.0150
3	0.0309	0		0.0148	0.0285	0.0209
4	0	0.0790	0.0903		0.0210	0.0095
5	0.4464	0.2218	0.1852	0.4892		0.0193
6	0.1181	0	0.0167	0.1558	0.0767	
