# Supplementary material for: High genetic diversity of spider species in a mosaic montane grassland landscape
Source: PLoS One. 2020 Jun 8;15(6):e0234437. doi: 10.1371/journal.pone.0234437 (PMC7279597; doi:10.1371/journal.pone.0234437)
Supplement: S3 Table — (PDF) [file pone.0234437.s004.pdf]

S3 Table. Diversity indices of *Neoscona subfusca* populations in the Golden Gate Highlands National Park, calculated from nucleotide sequence of the mitochondrial COI gene

Site	N	S	h	Hd	K	π
Site 1	12	60	10	0.9697	27.4546	0.0473
Site 2	12	52	8	0.8939	25.5455	0.0440
Site 3	11	51	5	0.8182	26.6546	0.0459
Site 4	11	56	7	0.8909	27.0909	0.0466
Site 5	12	59	10	0.9697	26.1970	0.0451
Site 6	10	16	5	0.7556	4.6000	0.0079
Total	68	64	17	0.9078	23.9723	0.0413

N: Number of sequences; S: Number of segregating (polymorphic/variable) sites; h: Number of haplotypes; Hd: Haplotype diversity; K: Average number of nucleotide differences; π: Nucleotide diversity.
