# Supplementary material for: High genetic diversity of spider species in a mosaic montane grassland landscape
Source: PLoS One. 2020 Jun 8;15(6):e0234437. doi: 10.1371/journal.pone.0234437 (PMC7279597; doi:10.1371/journal.pone.0234437)
Supplement: S4 Table — Fst values are represented in the bottom triangle of the matrix and Dxy values are represented in the top. (PDF) [file pone.0234437.s005.pdf]

S4 Table. Pairwise genetic differentiation (Fst) and nucleotide substitution per site (Dxy) among Golden Gate Highlands National Park populations of *Neoscona subfusca*. Fst values are represented in the bottom triangle of the matrix and Dxy values are represented in the top.

	1	2	3	4	5	6
1		0.0429	0.0433	0.0437	0.0431	0.0423
2	0		0.0416	0.0418	0.0416	0.0354
3	0	0		0.0428	0.0423	0.0389
4	0	0	0		0.0429	0.0394
5	0	0	0	0		0.0362
6	0.3476	0.2665	0.3088	0.3081	0.2685	
