# Supplementary material for: High genetic diversity of spider species in a mosaic montane grassland landscape
Source: PLoS One. 2020 Jun 8;15(6):e0234437. doi: 10.1371/journal.pone.0234437 (PMC7279597; doi:10.1371/journal.pone.0234437)
Supplement: S5 Table — (PDF) [file pone.0234437.s006.pdf]

S5 Table. Diversity indices of *Pherecydes tuberculatus* populations in the Golden Gate Highlands National Park, calculated from nucleotide sequence of the mitochondrial COI gene

Site	N	S	h	Hd	K	π
Site 1	10	0	1	0	0	0
Site 2	7	0	1	0	0	0
Site 3	12	1	2	0.4849	0.4849	0.0010
Site 4	12	1	2	0.4849	0.4849	0.0010
Site 5	3	0	1	0	0	0
Site 6	12	0	1	0	0	0
Total	56	2	3	0.2597	0.2701	0.0005

N: Number of sequences; S: Number of segregating (polymorphic/variable) sites; h: Number of haplotypes; Hd: Haplotype diversity; K: Average number of nucleotide differences; π: Nucleotide diversity.
