# Supplementary material for: High genetic diversity of spider species in a mosaic montane grassland landscape
Source: PLoS One. 2020 Jun 8;15(6):e0234437. doi: 10.1371/journal.pone.0234437 (PMC7279597; doi:10.1371/journal.pone.0234437)
Supplement: S6 Table — Fst values are represented in the bottom triangle of the matrix and Dxy values are represented in the top. (PDF) [file pone.0234437.s007.pdf]

S6 Table. Pairwise genetic differentiation (Fst) and nucleotide substitution per site (Dxy) among Golden Gate Highlands National Park populations of *Pherecydes tuberculatus*. Fst values are represented in the bottom triangle of the matrix and Dxy values are represented in the top.

	1	2	3	4	5	6
1		0	0.0007	0.0007	0	0
2	0		0.0007	0.0007	0	0
3	0.2727	0.2727		0.0013	0.0007	0.0007
4	0.2727	0.2727	0.2727		0.0007	0.0007
5	0	0	0.2727	0.2727		0
6	0	0	0.2727	0.2727	0	
