# Supplementary material for: High genetic diversity of spider species in a mosaic montane grassland landscape
Source: PLoS One. 2020 Jun 8;15(6):e0234437. doi: 10.1371/journal.pone.0234437 (PMC7279597; doi:10.1371/journal.pone.0234437)
Supplement: S7 Table — (PDF) [file pone.0234437.s008.pdf]

S7 Table. Diversity indices of *Philodromus browningi* populations in the Golden Gate Highlands National Park, calculated from nucleotide sequence of the mitochondrial COI gene

Site	N	S	h	Hd	K	π
Site 1	11	58	6	0.8364	11.9273	0.0310
Site 2	9	9	4	0.6944	2.4444	0.0064
Site 3	10	10	4	0.7111	2.6444	0.0069
Site 4	4	1	2	0.5000	0.5000	0.0013
Site 5	1	–	–	–	–	–
Site 6	–	–	–	–	–	–
Total	35	59	8	0.7112	5.2513	0.0136

N: Number of sequences; S: Number of segregating (polymorphic/variable) sites; h: Number of haplotypes; Hd: Haplotype diversity; K: Average number of nucleotide differences; π: Nucleotide diversity.
