# Supplementary material for: High genetic diversity of spider species in a mosaic montane grassland landscape
Source: PLoS One. 2020 Jun 8;15(6):e0234437. doi: 10.1371/journal.pone.0234437 (PMC7279597; doi:10.1371/journal.pone.0234437)
Supplement: S8 Table — Fst values are represented in the bottom triangle of the matrix and Dxy values are represented in the top. (PDF) [file pone.0234437.s009.pdf]

S8 Table. Pairwise genetic differentiation (Fst) and nucleotide substitution per site (Dxy) among Golden Gate Highlands National Park populations of *Philodromus browningi*. Fst values are represented in the bottom triangle of the matrix and Dxy values are represented in the top.

	1	2	3	4	5	6
1		0.0182	0.0183	0.0165	–	–
2	0		0.0060	0.0041	–	–
3	0	0		0.0043	–	–
4	0.0201	0.0702	0.0471		–	–
5	–	–	–	–		–
6	–	–	–	–	–	
