# Supplementary material for: High genetic diversity of spider species in a mosaic montane grassland landscape
Source: PLoS One. 2020 Jun 8;15(6):e0234437. doi: 10.1371/journal.pone.0234437 (PMC7279597; doi:10.1371/journal.pone.0234437)
Supplement: S9 Table — (PDF) [file pone.0234437.s010.pdf]

S9 Table. Diversity indices of the *Theridion* sp. populations in the Golden Gate Highlands National Park, calculated from nucleotide sequence of the mitochondrial COI gene

Site	N	S	h	Hd	K	π
Site 1	7	6	4	0.8095	2.2857	0.0047
Site 2	2	3	2	1	3.0000	0.0062
Site 3	4	3	3	0.8333	1.6667	0.0034
Site 4	12	11	7	0.9091	3.6061	0.0074
Site 5	5	3	2	0.6000	1.8000	0.0037
Site 6	3	4	3	1	2.6667	0.0055
Total	33	18	12	0.8807	3.1288	0.0064

N: Number of sequences; S: Number of segregating (polymorphic/variable) sites; h: Number of haplotypes; Hd: Haplotype diversity; K: Average number of nucleotide differences; π: Nucleotide diversity.
