# Supplementary material for: High genetic diversity of spider species in a mosaic montane grassland landscape
Source: PLoS One. 2020 Jun 8;15(6):e0234437. doi: 10.1371/journal.pone.0234437 (PMC7279597; doi:10.1371/journal.pone.0234437)
Supplement: S10 Table — Fst values are represented in the bottom triangle of the matrix and Dxy values are represented in the top. (PDF) [file pone.0234437.s011.pdf]

S10 Table. Pairwise genetic differentiation (Fst) and nucleotide substitution per site (Dxy) among Golden Gate Highlands National Park populations of the *Theridion* sp. Fst values are represented in the bottom triangle of the matrix and Dxy values are represented in the top.

	1	2	3	4	5	6
1		0.0063	0.0037	0.0084	0.0045	0.0052
2	0.1395		0.0051	0.0106	0.0056	0.0072
3	0	0.0667		0.0081	0.0037	0.0048
4	0.2765	0.3607	0.3269		0.0063	0.0061
5	0.0714	0.1111	0.0370	0.1186		0.0038
6	0.0189	0.1905	0.0714	0	0	
