# Supplementary material for: High genetic diversity of spider species in a mosaic montane grassland landscape
Source: PLoS One. 2020 Jun 8;15(6):e0234437. doi: 10.1371/journal.pone.0234437 (PMC7279597; doi:10.1371/journal.pone.0234437)
Supplement: S11 Table — (PDF) [file pone.0234437.s012.pdf]

S11 Table. Table indicating the dominant plant species present at the time of sampling in each site.

Dominant plant species	Site					
	1	2	3	4	5	6
Cussonia paniculata	X			X		
Diospyros whyteana	X			X	X	
Euclea crispa	X	X	X	X		
Gymnosporia buxifolia		X				
Olinia emarginata	X	X	X	X	X	X
Pittosporum viridiflorum	X	X	X	X		
Leucosidea sericea				X	X	X
Searsia dentata	X	X	X	X		
Olea europaea africana	X	X	X			
Widdringtonia nodiflora					X	X
Cliffortia linearifolia					X	X
Protea subvestita	X			X		
